# Protocol on the fabrication of monocrystalline thin semiconductor via crack-assisted layer exfoliation technique for photoelectrochemical water-splitting

**DOI:** 10.1016/j.xpro.2021.101015

**Published:** 2022-01-07

**Authors:** Yonghwan Lee, Bikesh Gupta, Hark H. Tan, Chennupati Jagadish, Jihun Oh, Siva Karuturi

**Affiliations:** 1 Convergence Materials Research Center, Gumi Electronics and Information Technology Research Institute (GERI), Gumi 39171, Republic of Korea; 2 Department of Electronic Materials Engineering, Research School of Physics, The Australian National University, Canberra, ACT 2601, Australia; 3 Australian Research Council Center of Excellence for Transformative Meta-Optical Systems, Research School of Physics, The Australian National University, Canberra, ACT 2601, Australia; 4 Department of Materials Science and Engineering, Korea Advanced Institute of Science and Technology (KAIST), Daejeon 34141, Republic of Korea; 5 School of Engineering, The Australian National University, Canberra, ACT 2601, Australia

**Keywords:** Physics, Energy, Chemistry, Material sciences

## Abstract

Thin semiconductors attract huge interest due to their cost-effective, flexible, lightweight, and semi-transparent properties. Here, we present a protocol on the preparation of thin semiconductor via controlled crack-assisted layer exfoliation technique. The protocol details the fabrication procedure for producing thin monocrystalline semiconductors with thicknesses in the range of a few tens of micrometers from thick donor substrates. In addition, we describe proof-of-concept application of the thin semiconductors for photoelectrochemical water-splitting to produce hydrogen fuel.

For complete details on the use and execution of this protocol, please refer to Lee et al. (2021).

## Highlights

• Protocol to obtain thin silicon (Si) via controlled crack-assisted layer exfoliation
• Free-standing thin Si with thickness in the range of few tens of micrometers
• Procedure for thin Si-based photoelectrochemical (PEC) water-splitting cells
• Troubleshooting the crack-assisted layer exfoliation process to obtain high yield

## Graphical abstract

**Figure:**
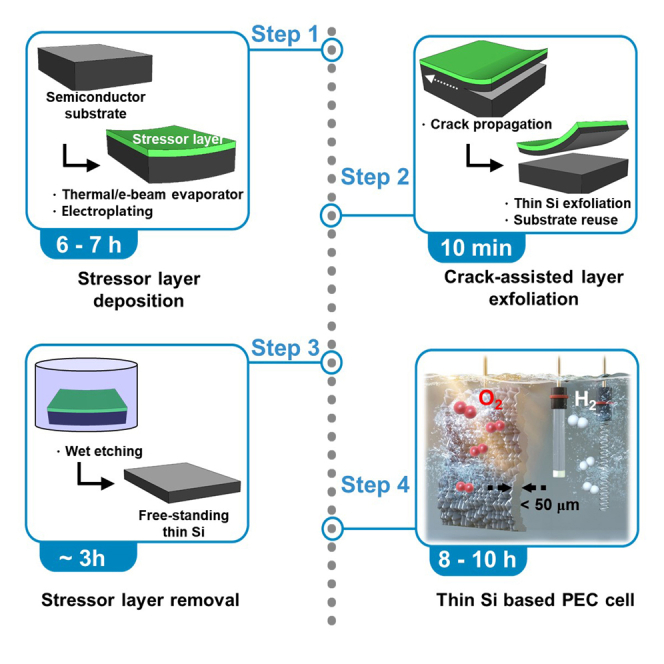

## Before you begin

Crack-assisted layer exfoliation method, which is also known as spalling process, can be used to produce thin semiconductor (< 50 μm), which is not possible by the standard wafering method used in current industry. This approach is achieved by controlling the sub-surface crack propagation direction in brittle semiconductor donor substrates. It is not only simple and cost-effective, but also applicable to any brittle substrate such as Si, Ge, GaAs, GaN and InP. A monocrystalline semiconductor donor substrate serves as a starting material to produce the thin semiconductors. In this protocol, we used commercial mirror-polished 500 μm-thick n-type Czochralski (CZ) crystalline Si wafers (phosphorus doped, ρ = 1–10 Ω·cm) as a starting material. However, we also previously used this protocol in other monocrystalline semiconductor substrates such as GaAs and InP.

Further knowledge on thin semiconductor exfoliation procedure can be found in the literature (Lee et al., 2020, 2021).

Refer to “materials and equipment” section for the list of equipment needed for this protocol.

### Preparation of nickel electroplating solution

**Timing: 1 day**

A number of approaches can be used to form the stressor layer on the Si donor substrate. For instance, sputter and electroplating processes are generally used in the crack-assisted layer exfoliation method (Lee et al., 2020). In this protocol, we used the electroplating process due to the highly controllability of the stress level and high deposition rates in a simple solution-based processing, whereas the sputter process demands complex vacuum processing technology. In the electroplating process, the residual stress of the deposited Ni stressor layer is strongly affected by the composition of electroplating solution. Especially, NiCl_2_ solution is known to form Ni thin films with high residual stress. In the following steps, we describe the preparation of NiCl_2_ based electroplating solutions.

1. Dissolve 22 g of H_3_BO_3_ powder into 600 mL of deionized (DI) water (obtained using Millipore DI water purification system) in a glass beaker under magnetic stirring. Stirring should be continued until the solution turns transparent without any precipitates.
2. Add 86 g of nickel chloride hexahydrate (NiCl_2_·6H_2_O) into the mixed solution under constant magnetic stirring for more than 12 h until the solution turns green without any undissolved precipitates. Use parafilm to cover the glass beaker containing the mixed solution to avoid water evaporation.

## Key resources table

**Table:** 

REAGENT or RESOURCE	SOURCE	IDENTIFIER
**Chemicals, peptides, and recombinant proteins**		

Monocrystalline Si substrate (n-type, 500 μm)	Semiconductor wafer, Inc (SWI), Taiwan	N/A
Ammonium hydroxide (NH_4_OH, 30%)	Chem Supply	CAS: 1336-21-6
Hydrogen peroxide (H_2_O_2_, 30%)	Chem Supply	CAS: 7722-84-1
Hydrogen fluoride (HF, 48%)	Merck	CAS: 7664-39-3
Hydrogen chloride (HCl, 36%)	Ajax Finechem	CAS: 7647-01-0
Nickel chloride hexahydrate (NiCl_2_·6H_2_O)	Sigma-Aldrich	CAS: 7791-20-0
Boric acid (H_3_BO_3_)	Sigma-Aldrich	CAS: 10043-35-3
Encapsulation epoxy	Henkel	LOCTITE EA 9460™
In-Ga eutectic alloy	Sigma-Aldrich	Catalog #: 495425

**Software and algorithms**		

CH Instruments Software	CH Instruments	https://www.chinstruments.com
ImageJ	Open source	https://imagej.net

**Other**		

Platinum counter electrode for Ni electrodeposition	neoscience	Catalog #: A-002250
Teflon tape	Saint-Gobain	Part no: 214338A5
Cold laminating pouch film	Local vendor	N/A
Punch with circular opening	EK Success	SKU: ek-54-20061
Ag/AgCl electrode	neoscience	Catalog #: A-012167
Platinum counter electrode for water-splitting measurement	neoscience	Catalog #: A-002234
E-beam evaporator	Ferrotec Temescal Systems	Product ID:BJD-2000
Potentiostat	CH Instruments	Product ID:CHI660E
Solar simulator	Abet Technologies	SunLite™ Solar Simulator 100 watt (Product ID: 11002-2)

## Materials and equipment

The Equipment needed:

• Instruments (for crack-assisted layer exfoliation)
  E-beam evaporator
  Potentiostat
• Instruments (for water-splitting performance measurement)
  Potentiostat
  Solar simulator

***Alternatives:*** In this protocol, we utilize an e-beam evaporator to deposit Ti and Ni seed layer on the Si donor substrate, however, alternative tools such as thermal evaporator can also be used.

## Step-by-step method details

### Wafer cleaning

**Timing: 30 min**

The first step for the crack-assisted layer exfoliation method involves a cleaning process of the Si donor substrate which is used as starting material to produce Si thin films. Conventional RCA standard clean 1 (RCA-1) cleaning process is used to remove organic residues on the surface of the Si donor substrate as below.

1. Prepare the RCA-1 solution with 20 mL of NH_4_OH (27%), 20 mL of H_2_O_2_ (30%), and 100 mL of DI water in a glass beaker.
2. Place the beaker on a hot plate and set the temperature to 40°C.
3. Place a Si donor substrate in the RCA-1 solution for 10 min (Some bubbles appear on the surface of the Si donor substrate in the RCA-1 solution)
4. Collect the Si donor substrate from the RCA-1 solution, and rinse it in copious amounts of DI water.
5. Collect the Si donor substrate from the DI water and blow-dry it using a nitrogen gun.

***Note:*** After the RCA-1 cleaning process, a native oxide layer is formed on the surface of the Si donor substrate. Therefore, the surface is modified into a hydrophilic surface. This can be confirmed by placing a water droplet and observing the shape of the water droplet on the Si donor substrate. Hydrophilic surface tends to be wetted by water droplets.

### Seed layer deposition on the cleaned Si donor substrate

**Timing: 3 h**

The following seed layer deposition process is an important step which leads to high adhesion between the Si donor substrate and Ni stressor layer deposited by electroplating process. To start the seed layer deposition process, the native oxide on the Si donor substrate should be removed via diluted HF solution. The removal of the native oxide on Si donor substrate enables high adhesion with the Ti seed layer. Ni stressor layer is immediately deposited on the Ti adhesion layer to avoid the oxidation of Ti layer upon atmospheric exposure. It is also noted that the unwanted Ti oxidation could also make it difficult to deposit Ni stressor layer via electroplating process due to an increased sheet resistance.

6. Prepare dilute HF solution (10%).

***Note:*** Teflon beaker should be used to prepare dilute HF solution to avoid the corrosion of glass beakers with the HF.

***Note:*** In conducting the processing with HF solution, careful attention to safety is required. HF readily penetrates the skin and dissociates into fluoride ions, and destructs deep tissue layers as well as bone. Personal protective equipment such as plastic face shield, acid resistant apron and gloves (i.e., neoprene rubber glove) are imperative while using HF solution.

7. Place the cleaned Si donor substrate in the diluted HF solution for 1 min to remove its surface native oxide.
8. Collect the Si donor substrate from the diluted HF solution and wash it with DI water.

***Note:*** The surface of Si donor substrate becomes hydrophobic after the removal of the native oxide by HF solution. The difference in the wetting property can be easily confirmed by placing a DI water droplet on the Si substrate.

9. Blow-dry the Si donor substrate with a nitrogen gun.
10. Additional air gun blowing steps can be used to remove any unwanted dust particles before placing the samples into the substrate holder for Ti evaporation.
11. Transfer the Si donor substrate to the e-beam evaporator chamber immediately.

**CRITICAL:** The Si donor substrate should be immediately loaded in the e-beam evaporator after native oxide removal to avoid the re-formation of native oxide in the ambient environment and possible contamination.

12. Control the rotation speed of the substrate holder at 50 rpm during the evaporation process.
13. When the vacuum reaches 5 × 10^−6^ mbar, the evaporation of the Ti/Ni seed layer is carried out as described below.
  a. The first 100 nm of Ti is evaporated at a deposition rate of 0.5 Å/s for pre-deposition without opening the sample chuck barrier.
  b. A 50 nm-thick Ti seed layer is deposited on the Si donor substrate at a deposition rate of 0.1–0.2 Å /s by opening the sample chuck barrier.
  c. In the next step, 50 nm of Ni is deposited on the Ti-coated Si donor substrate at a deposition rate of 0.1–0.2 Å/s.

***Note:*** Due to the pre-deposition of Ti layer, the vacuum level in the evaporator may be further improved. In our case, the vacuum improved to 10^−7^ mbar after Ti pre-deposition.

14. Allow time for cooling of the Si donor substrate after the seed layer deposition before collecting it from the sample loader.

### Stressor layer deposition

**Timing: 3 h**

For crack-assisted layer exfoliation method, high-tensile stressor film has to be deposited on the Si donor substrate. The stressor layer determines the crack initiation and crack propagation depth; therefore, it is important to determine the thickness and residual stress of the stressor layer. See the reference for detailed information on this (Lee et al., 2016).

15. Prepare the Ni electroplating solution as described above and place it under magnetic stirring for more than 1 h before commencing the electroplating process.
16. Cleave the seed layer-coated Si donor substrate to an appropriate size with diamond pencil.
17. Make a cold laminating pouch film-based shadow mask with a circular opening using a punching tool.
18. Attach the cold laminating pouch film with circular opening to the seed layer deposited Si donor substrate.
19. Fix the Si donor substrate to an electroplating zig and seal it with chemically inert teflon tape at the edge (see Figure 1 for our home-made electroplating zig).

**Figure 1:**
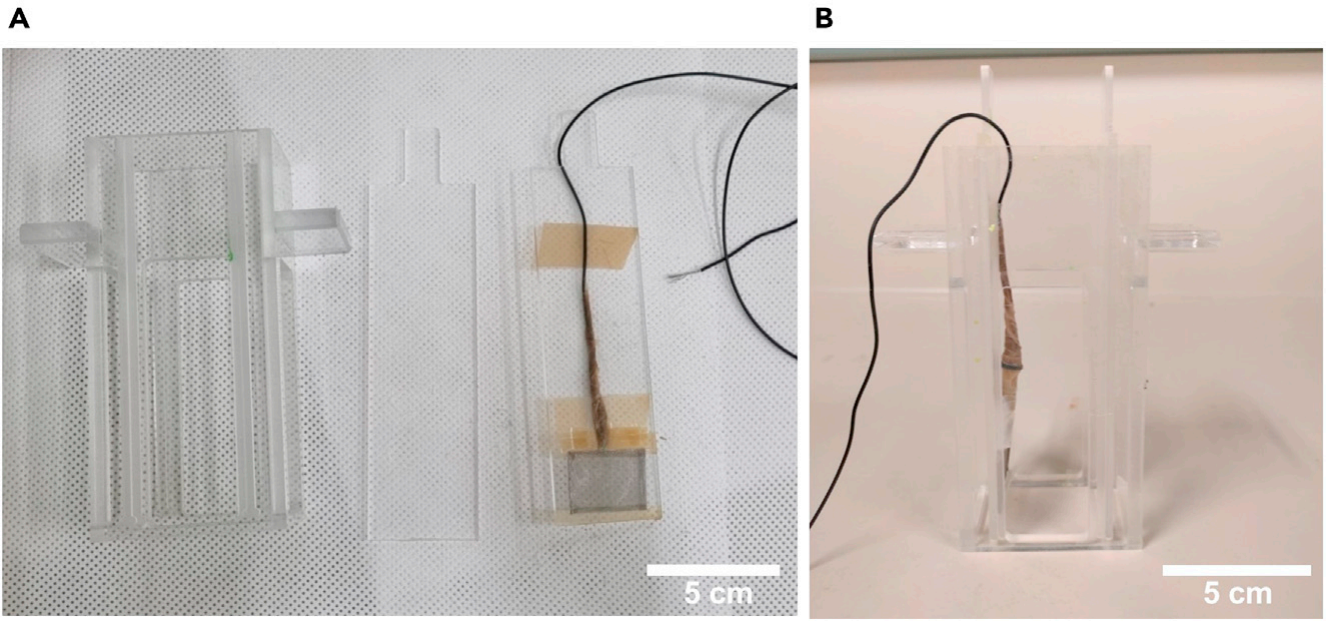
Home-made electroplating zig (A) Disassembled home-made electroplating zig. The part at the center is used to fix the Si donor substrate which is used as a working electrode. The far right part shows a mesh-type Pt counter electrode. (B) The assembled electroplating zig consisting of all the parts from (A).
20. Make an electrical contact to the seed layer deposited Si donor substrate using a copper wire and seal the exposed contact point and other open areas except for the circular opening area with teflon tape to protect from the electroplating solution (see Figure 2A).

**Figure 2:**
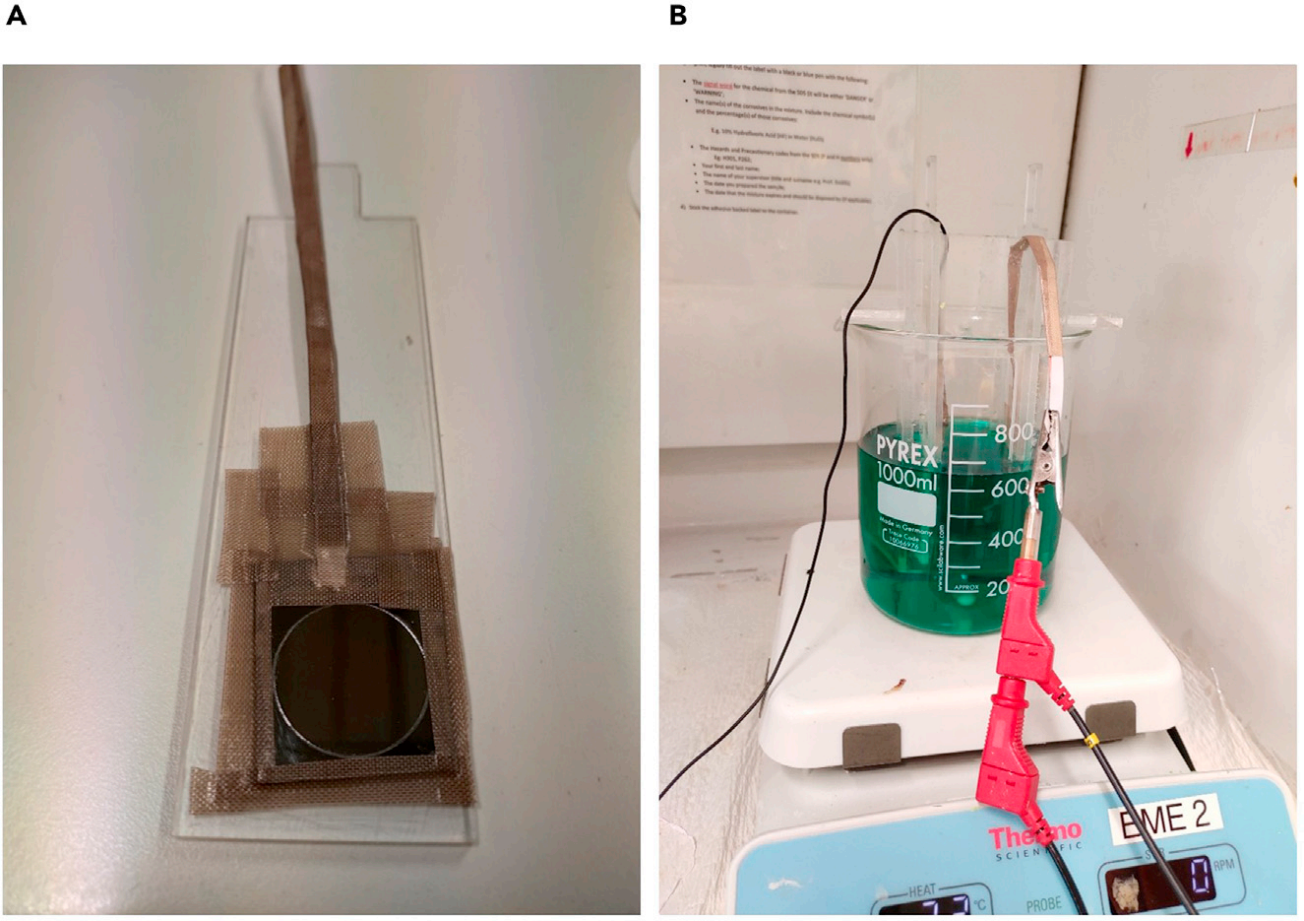
Set-up for Ni stressor layer deposition on seed layer-coated Si donor substrate (A) Seed layer-coated Si donor substrate with teflon tape at the edges. (B) Set-up for Ni stressor layer deposition with home-made electroplating set-up and Pt counter electrode. The electric wires contacting Si donor substrate and Pt counter electrode are connected to working and counter electrode terminals of the potentiostat, respectively.
21. Prepare dilute HCl solution (5%) and dip the sample in the solution for 5 min to remove the surface oxide on the Ni seed layer.
22. Take out the sample from the HCl solution and wash with DI water followed by blow drying using a nitrogen gun.
23. Dip the sample and a Pt counter electrode with home-made zig shown in Figure 1 in the as-prepared Ni electroplating solution.
24. Connect the electrical wire, which is attached to the seed layer-coated Si donor substrate, to the working electrode connection and the platinum counter electrode to the counter electrode connection of a potentiostat (see Figure 2B).

***Note:*** The electroplating process is conducted in traditional 2-electrode configuration using a potentiostat.

25. Use chronopotentiometry module with a constant operating current density of −20 mA/cm^2^ to electroplate the Ni stressor layer on the seed layer coated Si donor substrate.

***Note:*** The value of the operating current density at which the electroplating process is conducted must be carefully optimized to achieve the thickness uniformity and optimal residual stress. Generally, a high residual stress and low thickness uniformity of the electroplated Ni stressor layer occur at high operating current densities.

**CRITICAL:** At the start of the electroplating process, it is important to make sure the measured potential at pre-specified constant operating current density is sustained. The measured potential tends to increase steeply at the beginning and then starts to decrease gradually and saturate. If the potential continues to increase dramatically and surpasses the limits of the potentiostat system, it indicates a failure of the electroplating process. If this occurs, halt the electroplating process and assess the underlying problem. Some of the cases are described in the troubleshooting section.

**CRITICAL:** The exfoliated thickness of thin Si from donor substrate via crack-assisted exfoliation method is determined by the thickness of the stressor layer. Therefore, a precise control of the electroplating time, which in turn decides the thickness of the deposited Ni stressor layer, is necessary.

26. Take out the sample from the Ni electroplating solution after the completion of the electroplating process and then wash it thoroughly using DI water followed by blowing it dry with a nitrogen gun.
27. Remove the electrical wire connection and teflon tape which is used to seal and insulate the seed layer coated Si donor substrate.
28. Repeat steps 17–25 to prepare as many samples as needed.

***Note:*** The used Pt counter electrode can be cleaned with diluted HCl solution followed by DI water after the electroplating process.

**CRITICAL:** The seed layer-coated Si substrate and Pt counter electrode must be maintained at a constant distance with parallel alignment. Any inconsistency with this, the Ni electroplating rate changes due to changes in cell resistances during the electroplating process. It makes it difficult to achieve thickness control of the Ni stressor layer and alters the crack initiation position. Note that a distance of 4 cm between the seed layer-coated Si substrate and the Pt counter electrode was applied in this experiment considering the electrode areas (e.g., diameter of 2–3 cm) and stressor layer uniformity.

**CRITICAL:** The teflon tape has to be carefully wrapped around the electrical wire connection and seed layer-coated Si donor substrate. If electrolyte seeps in, it can lead to the loss of control over the area on which Ni stressor layer is deposited and also induces reduced Ni stressor layer on the targeted area.

### Crack-assisted layer exfoliation method

**Timing: 10 min**

**Figure 3:**
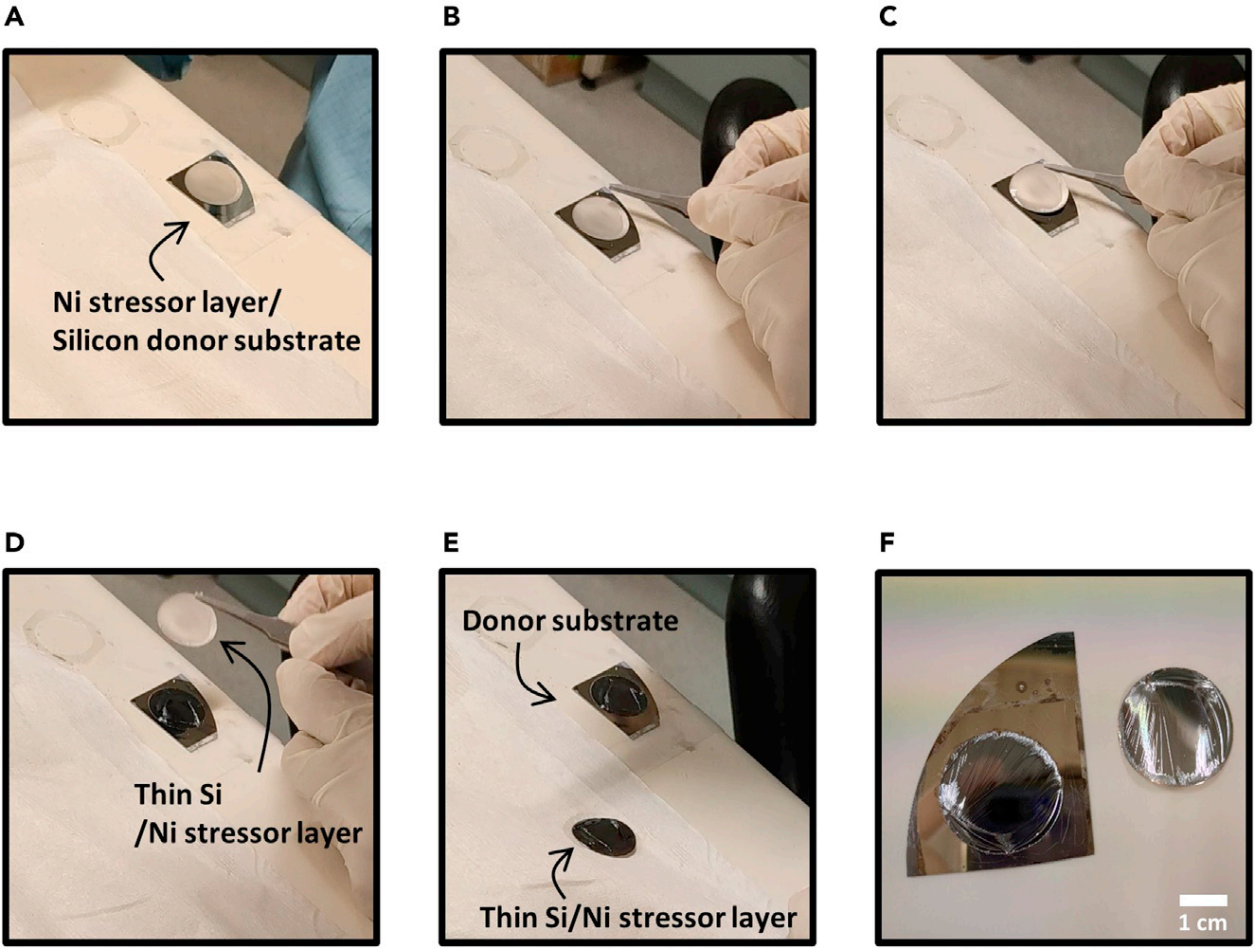
Photographic images illustrating the crack-assisted layer exfoliation to separate thin Si from the Si donor substrate (A) Grab the partially exfoliated Si from the initial crack at the edge of Ni stressor layer using a tweezer. (B) Manually apply force with a tweezer for crack propagation. (C) Fully exfoliate thin Si from the donor substrate. (D and E) Exfoliated donor substrate and thin Si with Ni stressor layer. (F) A magnified view of (E).

Initial crack is formed in the Si donor substrate after the stressor layer deposition if the stressor layer has sufficient thickness. The initial crack at/near the stressor layer can be observed by naked eye. The initial crack can be further grown by applying external force or thermal annealing process. In this protocol, we describe the initial crack propagation by applying external force to exfoliate Si thin film from its donor substrate (Figure 3). Refer to our past work for a detailed explanation on crack propagation using various approaches (Lee et al., 2020, 2021).

29. Fix the Ni stressor layer deposited Si donor substrate on a flat surface such as lab bench by attaching scotch tape at the edges of the Si donor substrate (Figure 3A).
30. Grasp the locally exfoliated thin Si with Ni stressor layer using a tweezer and manually pull it in vertical direction (Figures 3B and 3C).

***Note:*** Curved sharp tip tweezer enables an easy control of the crack propagation in the Si donor substrate while manually applying the mechanical force.

**CRITICAL:** The stressor layer must be thicker than the critical thickness for crack initiation in the Si donor substrate. The thickness of stressor layers can be increased with longer operating time and high operating current density in the electroplating process. See the reference for the more details (Lee et al., 2016).

31. Continue applying the external force until the crack propagates to the opposite edge of the initial crack position, and the Si thin film with Ni stressor layer is fully exfoliated from the Si donor substrate (see Figures 3D–3F).

**CRITICAL:** The crack propagation process is conducted with manually applied external force. In that process, the crack propagation must be conducted with a constant pulling speed that is not very abrupt. The surface roughness on the fracture surface increases if the crack propagation is carried out with high pulling force.

### Stressor layer removal

**Timing: 3 h**

The stressor layer is used for sub-surface crack propagation to exfoliate thin Si from its donor substrate. The stressor layer also can be useful as the mechanical handling layer which helps to prevent breaking of the fragile thin Si. However, the stressor layer needs to be removed in some cases after the crack-assisted layer exfoliation method. For instance, the stressor layer can limit the further processing of the spalled thin films in the high temperature annealing process and the conventional CMOS-compatible fabrication process. Therefore, the stressor layer has to be properly removed without causing damage on the exfoliated thin Si. In this protocol, we described the wet-etching approaches to remove the electroplated Ni stressor layer.

32. Prepare an etch solution for the stressor layer by mixing 20 mL of HCl (32%), 20 mL of H_2_O_2_ (30%), and 100 mL of DI water in a glass beaker.
33. Place the glass beaker with the mixed solution on a hot plate and set the temperature to 80°C.

***Note:*** Bubbles start appearing in the mixed solution when the temperature increases to 80°C. If bubbles do not appear in the mixed solution, add additional H_2_O_2_ solution (∼ 20 mL) to the mixed solution.

34. Immerse the sample in the mixed solution with dipper and lid to remove the Ni stressor layer on exfoliated thin Si.

***Note:*** The exfoliated thin Si can easily move in the beaker during the wet-etching process due to the formation of bubbles in the mixed solution. Dipper and lid can limit the movement of the exfoliated thin Si and helps to control the fragile thin Si after stressor layer removal.

**Figure 4:**
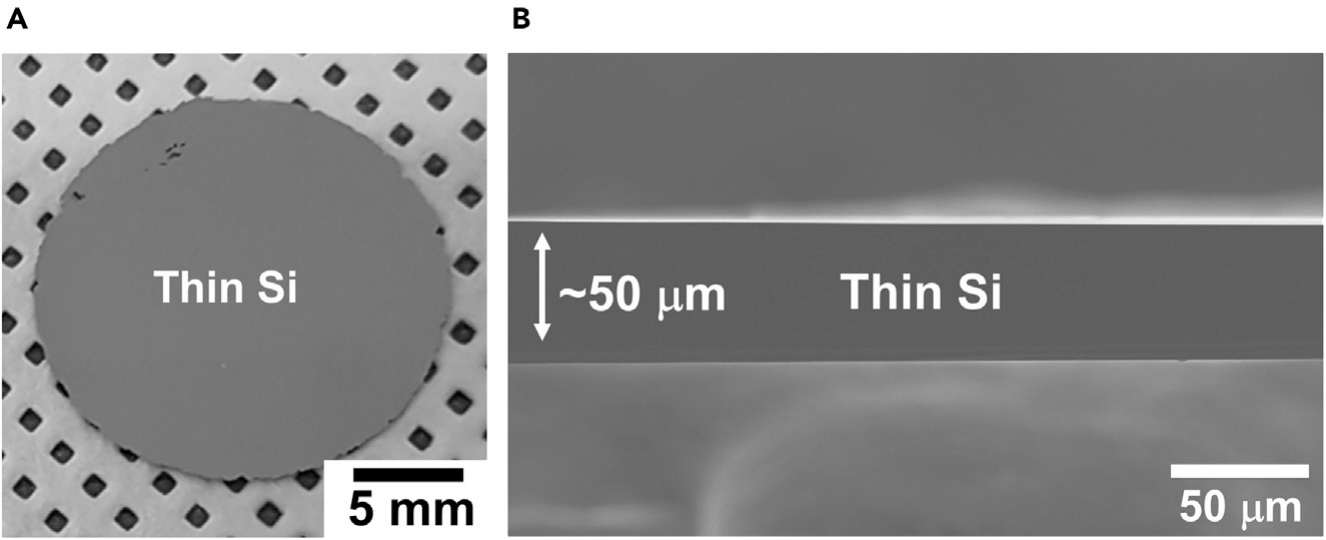
Free-standing thin Si after Ni stressor layer removal (A) Photograph of the thin Si (50 μm thick) after Ni stressor layer removal. (B) Cross-sectional SEM image of the thin Si. Figures were adopted with minor change from Lee et al. (2021).

35. Take out the sample from the mixed solution when the Ni stressor layer is fully removed and wash the remaining thin Si with DI water followed by drying under a gentle nitrogen flow (see Figure 4).

### Surface texturing of the thin Si

**Timing: 3 h**

Surface texturing on Si is a frequently used method to reduce the optical reflection at the surface for high performance solar conversion devices. Generally, micrometer sized random pyramid structure formation on the Si substrate with KOH solution is a standard procedure in Si processing. The wet-etching based surface texturing method is described below. It is noted that a cleaning process to remove residual potassium should also be accompanied due to the surface potassium induced critical performance degradation on the devices. The surface texturing process on thin Si also removes any surface defects formed after the crack-assisted exfoliation method. See the reference for more detailed information (Lee et al., 2018).

36. Dissolve 3 g of KOH pellets in 200 mL of DI water in a glass beaker under constant magnetic stirring until the solution turns transparent without any precipitates.
37. Place the glass beaker with the mixed solution on a hot plate and set the temperature to 80°C
38. Add 15 mL of isopropyl alcohol (IPA) into the mixed solution.
39. Place the thin Si in a dipper and close with the lid.
40. Dip the dipper with thin Si in the mixed solution for 40 min.

***Note:*** The thin Si can easily move in the mixed solution during the wet-etching process due to vigorous bubble generation at high temperature (∼80°C). Placing a lid for the dipper could limit the movement of the thin Si.

41. Take out the dipper with thin Si and wash with DI water.

***Note:*** After surface texturing, the color of the thin Si changes to gray due to the formation of micrometer size random pyramids on the surface.

42. Mix 20 mL of HCl (32%), 20 mL of H_2_O_2_ (30%), and 100 mL of DI water in a glass beaker.

***Note:*** The mixed solution to remove surface residual potassium after the surface texturing process is the same as the solution used for Ni stressor layer removal.

**Figure 5:**
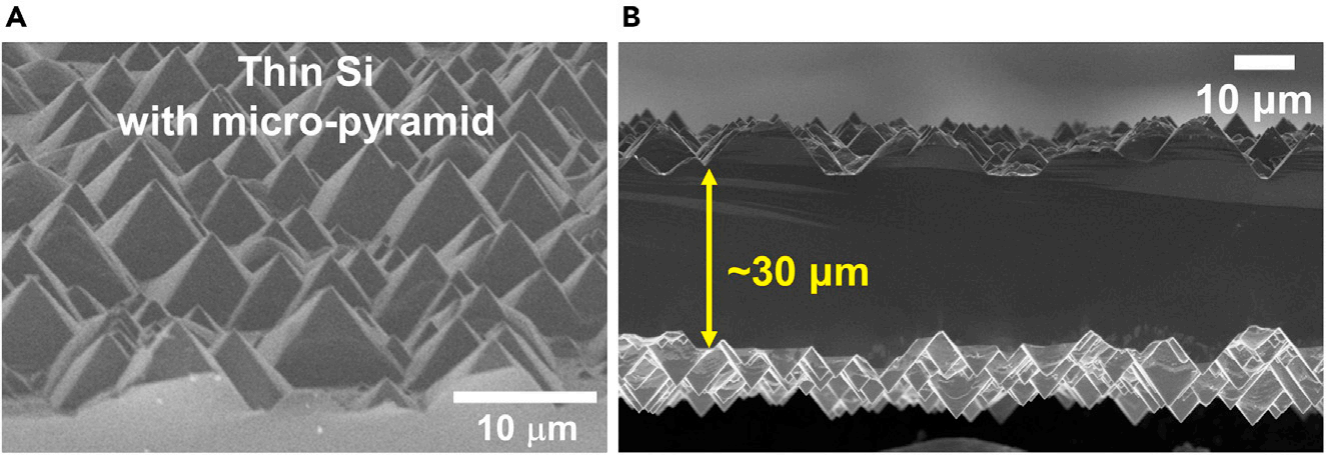
Micrometer-scale random pyramid structure on the thin Si (A and B) (A) Tilted and (B) cross-sectional SEM images of random pyramid structures formed on the thin Si via wet-etching process. Figures were adopted with minor change from Lee et al. (2021).

43. Place the glass beaker with the mixed solution on a hot plate and set the temperature to 80°C.
44. Place the sample on the dipper with a lid and immerse them in the mixed solution for 10 min to remove residual potassium.
45. Take out the dipper with thin Si from the mixed solution and wash with DI water followed by drying it under gentle nitrogen flow (see Figure 5).

### Fabrication of NiO_x_-coated thin Si photoanodes

**Timing: 8–10 h**

**Figure 6:**
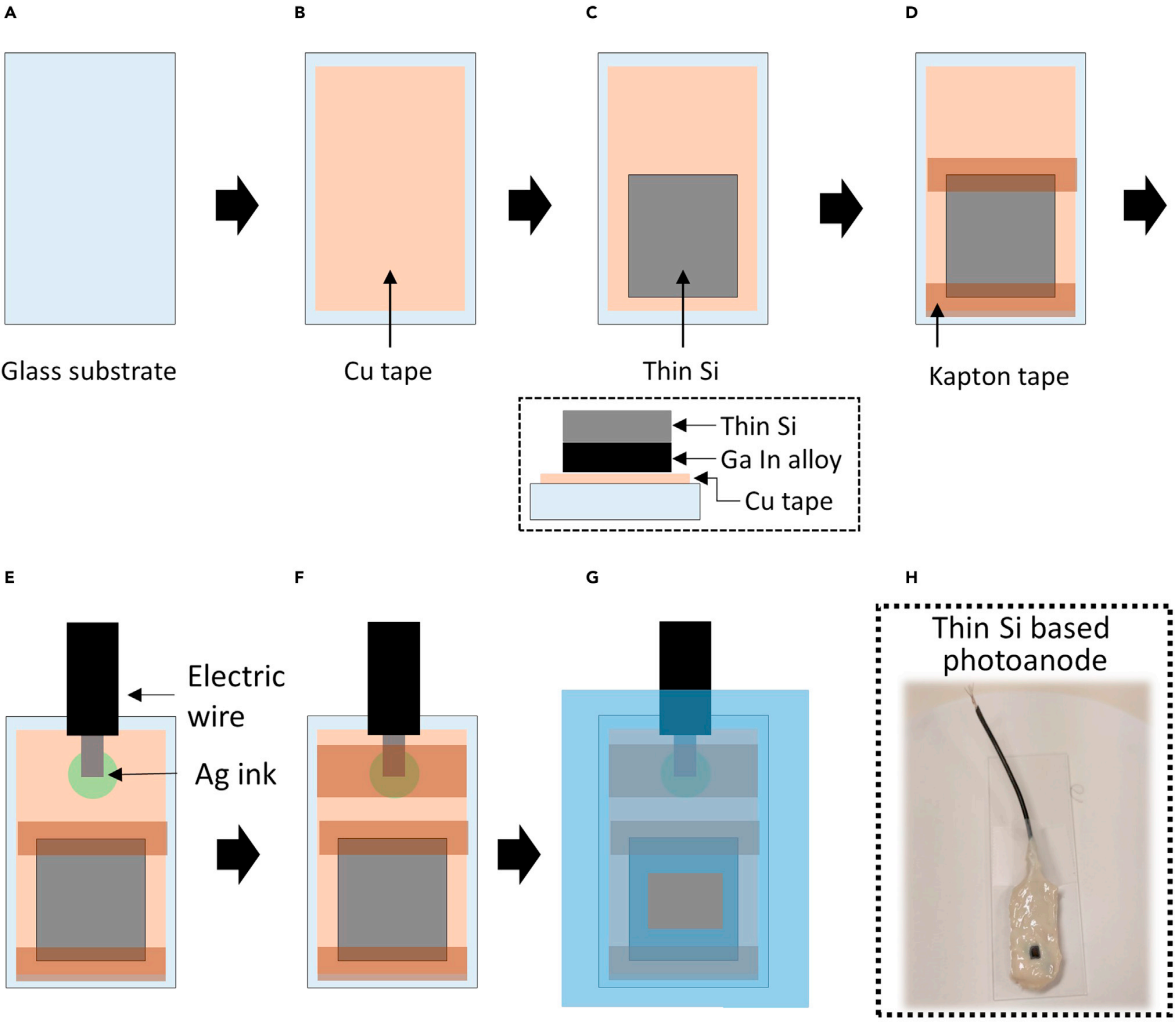
Assembling of thin Si based electrode for photoelectrochemical water-splitting cells (A–H) (A–G) Schematic illustration of the thin Si based photoanode and (H) photograph of the final fabricated electrode.

Co-catalyst deposition is important to enhance the performance for water-splitting using thin Si. However, device fabrication with thin Si requires careful handling due to the fragile nature of thin Si. Here, we describe the procedure for depositing NiO_x_, which is one of the commonly used oxygen evolution reaction (OER) co-catalysts, on the surface textured thin Si without creating additional damages. Figure 6 shows the illustrated detail procedure and fabricated NiO_x_-coated thin Si photoanode.

46. Prepare dilute HF solution (5%).
47. Immerse the dipper with thin Si in the diluted HF solution for 1 min to remove the native oxide on Si surface and wash with DI water followed by drying under gentle nitrogen flow.
48. Fix the textured thin Si on the rigid glass substrate by attaching Kapton tape at the edges of the thin Si.

***Note:*** The thin Si has a high likelihood of fracturing risk from mechanical shock during the handling process. Fixing the fragile thin Si on a rigid substrate helps to improve the processing yield by alleviating the mechanical stress during the fabrication process.

49. Place the sample in the sputter chamber and deposit 20 nm-thick NiO_x_ film on the textured surface via the DC sputtering process in the Ar atmosphere using NiO_x_ target.
50. Unload the sample and remove the attached Kapton tape at the edges of the sample.
51. Prepare the Cu tape attached glass substrate (Figure 6B).
52. Drop a tiny amount of liquid indium-gallium (InGa) alloy on the Cu tape and place the uncoated side of the sample on the InGa alloy to form an Ohmic contact. (Figure 6C)
53. Seal the NiO_x_-coated thin Si at the edges using Kapton tape (Figure 6D).
54. Form an electrical contact on Cu tape by connecting it to a wire with conductive Ag ink and drying it for 1 h in the fume hood followed by sealing with Kapton tape (Figures 6E and 6F).
55. Seal off the sample using chemically inert epoxy (Loctite 9460 or 9462) only exposing the area of the NiO_x_-coated front surface to protect it from corrosion in electrolyte during water splitting measurements (Figure 6G).
56. Dry the epoxy-coated sample on a hot plate or in an oven at 60°C for 2–3 h.

***Note:*** Place the epoxy-coated samples on a Kapton tape for baking when placing on a hot plate to avoid any undried epoxy gripping to the bottom surface.

### Measurement of photoelectrochemical water-splitting performance

**Timing: 1 h**

**Figure 7:**
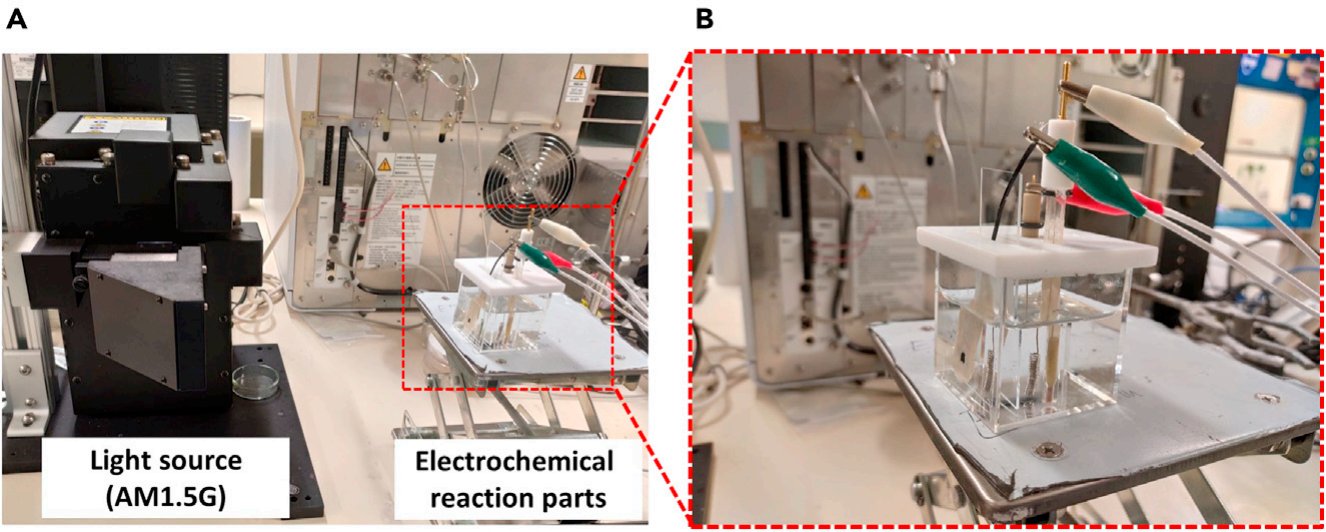
Photoelectrochemical water-splitting measurement set-up (A) Photograph of the light source (AM1.5G) and the electrochemical cell for water splitting measurement. (B) Closer view of the electrochemical cell with the thin Si photoanode, Ag/AgCl, and coil-type Pt electrode as working, reference, and counter electrode, respectively.

In this protocol, we describe the photoelectrochemical water-splitting performance measurement method of NiO_x_-coated thin Si photoanodes via conventional 3-electrodes configuration using a potentiostat. A coil-shaped Pt and Ag/AgCl (3M NaCl) electrode are used as a counter and reference electrodes, respectively. A 300 W Xe lamp equipped with AM 1.5G filter is used as the light source. The light intensity is set to 1 sun using a reference Si solar cell (see Figure 7).

57. Prepare 1 M KOH solution to be used as an electrolyte by mixing 14 g of KOH pellets and 250 mL of DI water under continuous magnetic stirring.
58. Pour the 1 M KOH solution into a quartz cell with a flat window for light exposure.
59. Set the conventional 3-electrode configuration measurement in the quartz cell filled with 1M KOH solution. The NiO_x_-coated thin Si based photoanode, coil-type Pt electrode, Ag/AgCl electrode are used as working electrode, counter electrode, and reference electrode, respectively.
60. Illuminate the quartz cell with a light source calibrated with 1 sun intensity at the surface of the quartz cell.
61. Measure the photoelectrochemical polarization curves in the anodic direction via linear sweep voltammetry method at a scan rate of 20 mV/s.
62. Take out the sample and wash with DI water followed by drying under gentle nitrogen flow using a nitrogen gun.

***Note:*** The working area of the NiO_x_-coated thin Si photoanode can be accurately calculated using a free image analysis software (ImageJ).

## Expected outcomes

This protocol provides the procedure for the exfoliation of monocrystalline thin Si with controlled thickness via sub-surface crack propagation. In this regard, we deposited the Ni stressor layer by controlling the thickness and stress level on the Si substrate. After Ni stressor layer deposition, a crack was initiated and propagated by an applied external force in the Si donor substrate. After that, thin Si (< 50 μm) was exfoliated from the Si donor substrate without material loss. In addition, the free-standing thin Si which is compatible with conventional CMOS fabrication process was also demonstrated after a well-controlled Ni stressor layer removal process.

For photoelectrochemical water-splitting application, surface texturing and NiO_x_ co-catalyst deposition were conducted on the thin Si. The resulting thin Si based photoelectrochemical cell exhibited an on-set potential and saturated photo-generated current density of 1.39 V and 23.8 mA/cm^2^, respectively. The capability for thin Si production not only adds flexibility to monocrystalline Si, but also results in cost-reduction from reduced material usage.

## Limitations

Thin Si with sub-50 μm thickness can be made via the crack-assisted layer exfoliation method. However, the thin Si is very vulnerable to external mechanical shocks and may result in low mechanical robustness. Therefore, careful handling of thin Si layers is necessary. Supporting substrates such as glass can also help to eliminate mechanical stress and shock during additional fabrication steps.

Although the crack assisted layer exfoliation method was demonstrated on thin monocrystalline semiconductors with sub 50 μm thickness, it was not realized on sub μm thickness. More delicate control and theoretical study are required to fabricate the sub μm thickness control via crack assisted layer exfoliation method.

Thin Si generally show low photo-current density compared to the thick Si (∼200 μm) due to low light absorption of Si compared to direct bandgap materials. This issue can be solved by surface texturing at the front and rear to induce light trapping effects. See the reference for more detailed information (Massiot et al., 2020).

## Troubleshooting

### Problem 1

The separation of the electroplated Ni stressor layer and seed layer from the Si donor substrate after the electroplating process (step 25 in “stressor layer deposition”).

### Potential solution

The Ni stressor layer may exfoliate itself together with the seed layer without resulting in crack initiation or propagation in the Si donor substrate due to insufficient adhesion between Ti seed layer and the Si substrate. It is required to carefully control the deposition of the seed layer on the Si substrate via e-beam/thermal evaporator under high vacuum and clean chamber. Clean the vacuum chamber if it has high residual coatings. Avoid the vacuum chamber if metal oxides are frequently used as evaporating sources.

### Problem 2

The rise of the measured potential recorded by the potentiostat to unexpected high values after commencing the electrodeposition process. For instance, the measured potential drops to −10 V which is closer to the limitation of our potentiostat (step 25 in “stressor layer deposition”).

### Potential solution

The sharp increase in the measured potential to unexpected high values is due to the separation of electrical connections to either working or counter electrodes. In that case, electrical connections to the working and Pt counter electrodes with electrical wires must be carefully checked. If Cu wire is used as the electrical connection, the Cu wire can be etched away by the Ni electroplating solution if exposed.

### Problem 3

Partial or no exfoliation of thin Si at the Ni stressor layer deposited area during the crack-assisted exfoliation process (step 31 in “crack-assisted layer exfoliation method”).

### Potential solution

Cleaning of any dust sitting on the seed layer before Ni electroplating by blowing with a nitrogen gun and/or ultrasonic cleaning.

### Problem 4

High surface roughness on the fracture surface of thin Si and donor substrate after crack-assisted layer exfoliation method (step 31 in “crack-assisted layer exfoliation method”).

### Potential solution

The high surface roughness of the fractured surface is due to the crack propagation in a skewed manner. To solve this problem, crack propagation speed and direction have to be carefully controlled by applying a gentle external force. Automation equipment to exfoliate the thin Si with constant speed is also recommended as shown in Wie et al. (2018).

### Problem 5

The thin Si is vulnerable to breakage from external mechanical shock and stress due an extremely reduced substrate thickness and edge chipping after stressor layer removal (step 35 in “stressor layer removal”).

### Potential solution

The thin Si fabricated by crack-assisted layer exfoliation method frequently has edge chipping which leads to breakage of the thin Si. The edge chipping often works as the fracture originating point during additional fabrication processing. Cleaving and removal of the edge chipping before additional fabrication processes could help to increase the processing yield. As mentioned, attaching thin Si to rigid supporting substrates also helps to increase the processing yield during the additional fabrication processing.

## Resource availability

### Lead contact

Further information and requests for resources, reagents and methods should be directed to the lead contact, Yonghwan Lee (yhlee@geri.re.kr).

### Materials availability

This study did not generate any new unique reagents.

## Data Availability

Data and code would be made available upon request.
